# In Memoriam

**Published:** 1886-02

**Authors:** 


### In Memoriam.

☛ SPECIAL REQUEST TO EVERY MEMBER.—The Secretary *earnestly requests each member of the Association* to aid him *in filling* THE BLANKS in the following Roll of Deceased Members *by making the necessary inquiries* in their respective cities and counties, *and writing him at once* on obtaining the desired information.

| NAME. | POST-OFFICE. | COUNTY. | ADMITTED. | DIED. |
|---|---|---|---|---|
| Adams, C. B | Augusta | Richmond | 1868 | 1875 |
| Alexander, J. R | | Floyd | 1849 | |
| Arnold, R. D. (V.-P., P.) | Savannah | Chatham | 1849 | 1876 |
| Avery, J. C | Decatur | DeKalb | 1859 | 1873 |
| Banks, J. T. (V.-P., P.) | Spalding | Griffin | 1858 | 1880 |
| Barkwell, T. J | Hawkinsville | Pulaski | 1859 | |
| Battle, H. L | Russellville | Monroe | 1849 | |
| Bell, T. W | Busbyville | Houston | 1853 | |
| Bignon, H. A | Augusta | Richmond | 1852 | 1870 |
| Billing, S. A | Columbus | Muscogee | 1855 | 1872 |
| Black, R. C. (T.) | Augusta | Richmond | 1852 | 1860 |
| Bomar, B. F | Atlanta | Fulton | 1859 | |
| Boswell, S. J. (V.-P.) | Columbus | Muscogee | 1855 | |
| Bothwell, D. J | Vienna | Dooly | 1849 | |
| Bozeman, J. F | Atlanta | Fulton | 1855 | 1877 |
| Brandon, D. S | Thomasville | Thomas | 1873 | 1878 |
| Broadhurst, W. W | Augusta | Richmond | 1852 | 1861 |
| Bruce, R. J. | Thomasville | Thomas | 1874 | 1880 |
| Bunn, William | Wilna | Houston | 1854 | |
| Burgess, William R. (T., O.) | Macon | Bibb | 1870 | 1878 |
| Burney, S. W. (P.) | | | | 1876 |
| Campbell, Robert | Augusta | Richmond | 1849 | 1885 |
| Carroll, J. C | Laurens Hill | Laurens | 1857 | 1870 |
| Carroll, R. C | Augusta | Richmond | 1873 | 1882 |
| Castlen, F. G | Macon | Bibb | 1867 | 1874 |
| Carlton, J. M | Athens | Clarke | 1882 | 1883 |
| Charters, W. M. (P.) | Savannah | Chatham | 1851 | 1883 |
| Chase, D. S | Augusta | Richmond | 1852 | |
| Clark, S. B | Richmond Factory | Richmond | 1852 | |
| Coe, J. N | Flat Rock | Pike | 1856 | |
| Colley, F. S. (P.) | Monroe | Walton | 1856 | |
| Cooper, Geo. F. (C. S., V.-P.) | Americu's | Sumter | 1849 | 1882 |

| NAME. | POST-OFFICE. | COUNTY. | ADMITTED. | DIED. |
|---|---|---|---|---|
| Copeland, A. B | Hamilton | Harris | 1878 | 1882 |
| Cornwall, G. H | Hillsboro | Jasper | 1854 | |
| Crawford, S. S | Augusta | Richmond | 1857 | |
| Crump, E. D | Herndon | Burke | 1872 | 1881 |
| Cumming, H. M | Augusta | Richmond | 1868 | 1872 |
| Cunningham, L. S | Big Creek | Forsyth | 1859 | 1862 |
| Cunningham, W. D | Jasper | Pickens | 1859 | 1866 |
| D'Alvigney, N | Atlanta | Fulton | | 1877 |
| Davenport, C. W | Point Peter | Oglethorpe | 1852 | |
| Davenport, H. S | Calhoun | Gordon | 1859 | |
| Davis, W. L | Albany | Dougherty | 1849 | |
| Dean, S. H | Conyers | Rockdale | 1856 | |
| Dearing, W. E | Augusta | Richmond | 1852 | 1867 |
| DeCortez, C. A | Savannah | Chatham | 1872 | |
| Dickinson, J. T | Albany | Dougherty | 1857 | |
| Dickinson, R. Q. (V.-P., P.) | Albany | Dougherty | 1850 | |
| Dugas, L. A. (P.) | Augusta | Richmond | 1852 | 1884 |
| Dupree, L. J | Camilla | Mitchell | | |
| Eve, Jos. A | Augusta | Richmond | 1852 | 1886 |
| Eve, S. C | Augusta | Richmond | 1868 | 1884 |
| Felder, W. L | Augusta | Richmond | 1857 | 1867 |
| Fish, J. D. (T.) | Savannah | Chatham | 1875 | 1879 |
| Fitzgerald, E | Macon | Bibb | 1882 | 1885 |
| Ford, L. D | Augusta | Richmond | 1849 | 1884 |
| Fort, Moses T | Hawkinsville | Pulaski | 1859 | 1878 |
| Franklin, M. A | Macon | Bibb | 1849 | 1858 |
| Frazier, W. M | Hawkinsville | Pulaski | 1850 | |
| Gaither, B. T | Oxford | Newton | 1852 | 1853 |
| Gardner, J. W | Augusta | Richmond | 1857 | |
| Girardey, Edward | Augusta | Richmond | 1852 | 1859 |
| Gordon, J. M | Savannah | Chatham | 1849 | |
| Gray, S. H | Barnesville | Pike | 1871 | 1885 |
| Green, A. B | Pond Town | Sumter | 1849 | |
| Green, H. K | Macon | Bibb | 1849 | 1867 |
| Green, Thomas F | Milledgeville | Baldwin | 1849 | 1879 |
| Gregory, J. D | | Sumter | 1849 | |
| Grimes, T. W | Columbus | Muscogee | 1855 | 1882 |
| Habersham, J. C | Savannah | Chatham | 1853 | |
| Habersham, J. C., Jr. (V-P.) | Savannah | Chatham | 1866 | 1881 |
| Habersham, S. E | Eatonton | Putnam | 1870 | |
| Harden, W. P | Harmony Grove | Jackson | 1853 | 1885 |
| Harrell. W. J | Bainbridge | Decatur | 1874 | 1881 |
| Harris, S. N. (V.-P.) | Savannah | Chatham | 1851 | 1854 |
| Harrison, Gabriel | Macon | Bibb | | 1862 |
| Harriss, Juriah (O., V.-P.) | Savannah | Chatham | 1852 | 1876 |
| Hart, A. C | Waynesboro | Burke | 1852 | |

| NAME. | POST-OFFICE. | COUNTY. | ADMITTED. | DIED. |
|---|---|---|---|---|
| Hawes, E. C | Appling | Columbia | 1852 | |
| Heard, T. O | Griffin | Spalding | 1867 | |
| Hendrick, J. B | Covington | Newton | 1857 | 1881 |
| Henry, G. C | West Point | Troup | 1872 | |
| Hook, E. B | Augusta | Richmond | 1857 | 1862 |
| Hoxey, Thomas | Columbus | Muscogee | 1849 | 1853 |
| Hunter, E. H. W | Louisville | Jefferson | 1857 | 1883 |
| Ingraham, E. P | Albany | Dougherty | 1870 | 1873 |
| Irvine, Robert | Augusta | Richmond | 1880 | 1881 |
| Johnson, C. W | Macon | Bibb | 1870 | 1875 |
| Jones, B. O | Atlanta | Fulton | | |
| Jones, J. W | Atlanta | Fulton | 1857 | 1871 |
| Kirkscey, E. J. (V.-P.) | Columbus | Muscogee | 1871 | 1877 |
| Kollock, P. M. (P.) | Savannah | Chatham | 1852 | 1872 |
| Lamar, T. R. (V.-P.) | Macon | Bibb | 1849 | 1859 |
| Lightfoot, W. S | Macon | Bibb | 1849 | 1864 |
| Lockhart, R. H | Columbus | Muscogee | 1855 | 1859 |
| Long, Crawford W | Athens | Clarke | 1849 | 1878 |
| Lumpkin, George | Maxey's Depot | Oglethorpe | 1859 | |
| Lumpkin, S. P | Watkinsville | Clarke | 1859 | |
| Mackie, J. D | Augusta | Richmond | 1852 | 1854 |
| Maffit, R. G. W | Dalton | Whitfield | 1859 | |
| Mathews, W. D | Fort Valley | Houston | 1884 | 1885 |
| McBride, W. G | Oconee | Washington | 1850 | 1873 |
| McGoldrick, R | Macon | Bibb | 1849 | 1853 |
| McKinley, C. A | Newnan | Coweta | 1859 | |
| McMillan, J. E | Columbus | Muscogee | 1872 | 1883 |
| Means, A. (V.-P.) | Oxford | Newton | 1851 | 1883 |
| Means, O. S | Oxford | Newton | 1857 | |
| Meiere, William S | Madison | Morgan | 1857 | |
| Milam, R. J | Fairburn | Campbell | 1882 | 1882 |
| Morrison, D. H | Savannah | Chatham | 1868 | 1869 |
| Musgrove, W. C. (C. S.) | Midville | Burke | 1849 | 1876 |
| Nash, R. A | Marion | Twiggs | 1849 | |
| Nisbet, R. H | Eatonton | Putnam | 1854 | 1873 |
| Nottingham, C.B. (S., T., C., P.) | Macon | Bibb | 1849 | 1875 |
| Nunn, R. M | Savannah | Chatham | 1871 | 1873 |
| Ogilby, H. J. (V.-P.) | Madison | Morgan | 1849 | 1873 |
| O'Keefe, D. C. (S.) | Atlanta | Fulton | 1852 | 1871 |
| Orme, L. H. (S.) | Atlanta | Fulton | 1866 | 1872 |
| O'Sullivan, —— | Cork | Lee | 1849 | |
| Owen, J. D | | Baldwin | 1850 | |
| Parsons, J. M | Russellville | Monroe | 1849 | |
| Pitts, G. W | Star | Butts | 1859 | |
| Posey, J. F | Savannah | Chatham | 1851 | |
| Pringle, W | Covington | Newton | | |

| NAME. | POST-OFFICE. | COUNTY. | ADMITTED. | DIED. |
|---|---|---|---|---|
| Ragan, Isham H | Palmyra | Lee | 1856 | |
| Raines, Thomas | Atlanta | Fulton | 1872 | 1883 |
| Raines, T. L | Atlanta | Fulton | 1882 | 1884 |
| Rauschenberg, Christian | Atlanta | Fulton | 1861 | 1878 |
| Richardson, C. P | Savannah | Chatham | 1851 | 1853 |
| Ridley, R. A. T. (V-P.) | LaGrange | Troup | 1859 | 1871 |
| Robertson, J. J | Washington | Wilkes | 1852 | 1872 |
| Roddy, R. L. (V-P.) | Forsyth | Monroe | 1849 | 1878 |
| Ruffin, W. R | Augusta | Richmond | 1852 | |
| Russell, W. J | Lawrenceville | Gwinnett | 1861 | |
| Ryan, T. D. L | Hawkinsville | Pulaski | 1859 | 1876 |
| Safford, S. J | Madison | Morgan | 1856 | 1859 |
| Searcy, D. B | Bolingbroke | Monroe | 1849 | 1885 |
| Shropshire, J. W | | | 1871 | |
| Simmons, J. N | Atlanta | Fulton | 1850 | 1871 |
| Simmons, J. S | Gainesville | Hall | 1873 | 1881 |
| Simmons, S. B | Augusta | Richmond | 1852 | 1867 |
| Simmons, T. A | Irwinton | Wilkinson | 1871 | 1872 |
| Smith, A. D | Montezuma | Macon | 1871 | |
| Smith, C. W | Jonesboro | Clayton | 1859 | |
| Smith, R. M. (C.) | Athens | Clarke | | 1879 |
| Smith, Thomas | Savannah | Chatham | 1858 | 1876 |
| Spier, W. A | Grantville | Coweta | 1859 | |
| Stallings, J. D | Preston | Webster | 1871 | |
| Stanford, F. A | Columbus | Muscogee | 1849 | 1885 |
| Starr, E. P | Savannah | Chatham | 1868 | 1873 |
| Steele, H | Knoxville | Crawford | 1849 | |
| Steele, R. T | Fairburn | Campbell | 1849 | |
| Stephens, W. B | Forsyth | Monroe | 1849 | |
| Stewardson, Thomas | Savannah | Chatham | 1849 | |
| Stozier, L. L | Albany | Dougherty | 1867 | |
| Sutton, C. B | | Lee | 1856 | |
| Taylor, E. T | Columbus | Muscogee | 1855 | |
| Taylor, R. N | Hawkinsville | Pulaski | 1850 | |
| Thomas, J. G. (V-P., C., T.) | Savannah | Chatham | 1867 | 1885 |
| Thompson, Charles | Macon | Bibb | 1849 | 1854 |
| Trammell, A. A | Forsyth | Monroe | 1856 | |
| Underwood, J. B | Cave Springs | Floyd | 1860 | 1881 |
| Van Meter, J. N | Euharlee | Bartow | 1879 | 1881 |
| Wall, W. W | Calhoun | Gordon | 1860 | |
| Ware, A. E. O | | | 1859 | |
| Wells, C. H | Savannah | Chatham | 1853 | 1854 |
| West, Charles (P.) | Perry | Houston | 1849 | |
| West, C. W | Savannah | Chatham | 1853 | 1859 |
| White, S. G. (V-P.) | Milledgeville | Baldwin | 1868 | 1877 |
| Whitlock, Isaac W | Washington | Wilkes | 1852 | 1853 |

| Name. | Post-Office. | County. | Admitted. | Died. |
|---|---|---|---|---|
| Wiley, J. B. | Macon | Bibb | 1849 | |
| Williams, T. L. | | Dooly | 1849 | |
| Williams, W. L. | Fayetteville | Fayette | 1873 | 1878 |
| Willingham, Willis | Lexington | Oglethorpe | 1849 | 1872 |
| Winn, G. A. | Bolingbroke | Monroe | 1849 | 1862 |
| Wirsen, G. F. | Rutledge | Morgan | 1871 | 1884 |
| Woodson, C. T. | Wilna | Houston | 1854 | |
| Wooten, W. H. | Lexington | Oglethorpe | 1852 | 1860 |
| Wragg, J. A. | Savannah | Chatham | 1853 | |
| Yonge, Easton | Savannah | Chatham | | 1880 |

